# A Phosphorescent Iridium(III) Complex‐Modified Nanoprobe for Hypoxia Bioimaging Via Time‐Resolved Luminescence Microscopy

**DOI:** 10.1002/advs.201500107

**Published:** 2015-06-25

**Authors:** Wen Lv, Tianshe Yang, Qi Yu, Qiang Zhao, Kenneth Yin Zhang, Hua Liang, Shujuan Liu, Fuyou Li, Wei Huang

**Affiliations:** ^1^ Key Laboratory for Organic Electronics and Information Displays and Institute of Advanced Materials (IAM)Jiangsu National Synergetic Innovation Center for Advanced Materials (SICAM)Nanjing University of Posts and TelecommunicationsNanjing210023P.R. China; ^2^ Department of Chemistry and the State Key Laboratory of Molecular Engineering of Polymers and Institute of Biomedicine ScienceFudan UniversityShanghai200433P.R. China; ^3^ Key Laboratory of Flexible Electronics (KLOFE) and Institute of Advanced Materials (IAM)Jiangsu National Synergetic Innovation Center for Advanced Materials (SICAM)Nanjing Tech University (NanjingTech)Nanjing211816P.R. China

**Keywords:** biosensors, energy transfer, oxygen nanoprobes, phosphorescence, time‐resolved luminescent imaging

## Abstract

Oxygen plays a crucial role in many biological processes. Accurate monitoring of oxygen level is important for diagnosis and treatment of diseases. Autofluorescence is an unavoidable interference in luminescent bioimaging, so that an amount of research work has been devoted to reducing background autofluorescence. Herein, a phosphorescent iridium(III) complex‐modified nanoprobe is developed, which can monitor oxygen concentration and also reduce autofluorescence under both downconversion and upconversion channels. The nanoprobe is designed based on the mesoporous silica coated lanthanide‐doped upconversion nanoparticles, which contains oxygen‐sensitive iridium(III) complex in the outer silica shell. To image intracellular hypoxia without the interferences of autofluorescence, time‐resolved luminescent imaging technology and near‐infrared light excitation, both of which can reduce autofluorescence effectively, are adopted in this work. Moreover, gradient O_2_ concentration can be detected clearly through confocal microscopy luminescence intensity imaging, phosphorescence lifetime imaging microscopy, and time‐gated imaging, which is meaningful to oxygen sensing in tissues with nonuniform oxygen distribution.

## 1 Introduction

Oxygen plays a crucial role in many pathological and physiological processes in biological systems.^1^ It is the terminal acceptor of the electron transport chain, which supports cell metabolism and mitochondrial function.^2^ Hypoxia, often used to refer to an inadequate supply of oxygen, is one of the most important features of many diseases such as solid tumors, inflammatory diseases, and cardiac ischemia.^3^, ^4^, ^5^, ^6^ Hence, accurate monitoring of oxygen level in complicated biological systems is of paramount importance for disease diagnosis and therapy evaluation.

Recently, phosphorescent transition‐metal complexes, such as platinum(II) and palladium(II) porphyrins, and ruthenium(II) and iridium(III) complexes,^7^, ^8^, ^9^, ^10^ have been employed to sense and image the oxygen levels in living organisms in real time nondestructively and reversibly. The phosphorescence of these transition‐metal complexes can be quenched by oxygen molecules, owing to energy transfer caused by diffusion‐controlled collisional interaction between the triplet ground state of molecular oxygen and the long‐lived triplet excited state of the complexes.^11^, ^12^ However, these oxygen probes usually require ultraviolet (UV) or visible light excitation, which induces autofluorescence in biological systems.

Time‐resolved luminescence imaging technology (TRLI) can effectively eliminate the interferences of short‐lived autofluorescence from long‐lived phosphorescence.^13^, ^14^, ^15^, ^16^, ^17^, ^18^, ^19^, ^20^ It includes phosphorescence lifetime imaging microscopy (PLIM) and time‐gated luminescence imaging technology (TGLI). PLIM distinguishes the long‐lived phosphorescence signal from the short‐lived autofluorescence,^21^ whereas TGLI selectively collects long‐lived phosphorescence signal by exerting a delay time.^22^ Phosphorescent transition‐metal complexes are suitable for application in TRLI owing to their long emission lifetimes, although the reports about time‐resolved luminescent imaging of intracellular hypoxia were rare.^23^

Near‐infrared (NIR) light excitation is another method which could eliminate the generation of autofluorescence and increase light penetration depth into tissues. Phosphorescent complexes like platinum(II) and palladium(II) porphyrins could realize red light excitation by enlarge the conjugated structures.^7^, ^8^, ^11^ However it is difficult to extend the excitation wavelength to the NIR region. Upconversion luminescence (UCL) is a unique process where long‐wavelength photons are converted into short‐wavelength ones.^24^, ^25^ Rare‐earth‐based materials codoped with lanthanide show excellent UCL under continuous‐wave (CW) NIR excitation (typically 980 nm). Two‐photon excitation is another typical upconversion process. These two kinds of upconversion have been reported to be practical in biological oxygen sensing.^26^, ^27^, ^28^ Two‐photon oxygen probes were characterized by improved spatial confinement and reduced risk of photodamage,^26^ but usually require expensive femtosecond pump lasers and higher excitation power density (≈10^6^ W cm^−2^).^29^, ^30^ Lanthanide‐doped upconversion nanoparticles (UCNPs) can be excited by low‐cost CW NIR laser (typically 980 nm) with low excitation power density (≈10^2^ W cm^−2^),^31^, ^32^, ^33^ thereby eliminating most autofluorescence from biosamples and minimizing photodamage.^24^, ^25^, ^26^, ^27^, ^28^, ^29^, ^30^, ^31^, ^32^, ^33^, ^34^, ^35^, ^36^, ^37^, ^38^ These features render UCNPs good platform for designing NIR‐excitable oxygen probes.^29^, ^39^

**Figure 1:**
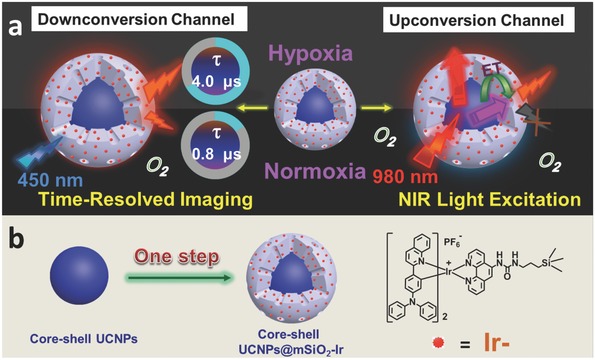
Schematic illustration of a) oxygen‐sensitive mechanism with downconversion channel and upconversion channel and b) synthesis of core–shell UCNPs@mSiO_2_‐Ir.

In this work, we have designed and synthesized an oxygen nanoprobe based on the mesoporous silica‐coated core–shell UCNPs covalently attached by complex **Ir**, denoted as core–shell UCNPs@mSiO_2_‐Ir (**Figure** **1**a). As for monitoring oxygen level under downconversion channel, the lifetime of oxygen sensitive long‐lived phosphorescence of the nanoprobe reduced from 4.0 to 0.8 μs when the environment transforms from hypoxia into normoxia. The change of phosphorescent lifetime can be visualized via phosphorescence lifetime imaging microscopy so that we can monitor oxygen level readily. Besides, short‐lived autofluorescence (several nanoseconds) can be eliminated via time‐gated luminescence imaging technology by exerting an appropriate delay time to enhance signal to noise ratio. As for upconversion channel, ultraviolet and blue UCL of UCNPs excited by 980 nm NIR light was served as energy source to excite complex **Ir** through energy transfer process, resulting in oxygen‐quenchable phosphorescence. Thus, NIR light excitable oxygen probe without autofluorescence can be also realized.

## 2 Results and Discussion

### 2.1 Synthesis and Characterization of Nanoprobe

The nanoprobe was prepared via the procedure shown in Figure 1b. First, oleic acid (OA) coated core–shell UCNPs (NaYF_4_: 20 mol% Yb/0.2 mol% Tm@NaYF_4_), which were prepared by a modified solvothermal process,^32^ were translated to water‐soluble core–shell UCNPs by hexadecyl trimethyl ammonium bromide (CTAB). Then, tetraethylorthosilicate (TEOS) and 3‐(triethoxysilyl)propyl isocyanate functionalized complex **Ir** were added to form the outer mesoporous silica shell on the core–shell UCNPs. In core–shell UCNPs@mSiO_2_‐Ir, the triplet excited state of complex **Ir** can be partially protected by the outer silica shell from excessive quenching by external environment. Therefore, the intensity of the phosphorescence is strong enough to ensure the accuracy of the collected emission lifetime data under a relatively high oxygen concentration, which is very important for time‐resolved luminescent imaging. NaYF_4_:Yb/Tm@NaYF_4_ UCNPs (core–shell UCNPs) with the higher upconversion efficiency than NaYF_4_:Yb/Tm UCNPs (core UCNPs) (Figure S1, Supporting Information) was chosen as energy donor to increase the energy transfer efficiency from UCNPs to complex **Ir**.^40^

**Figure 2:**
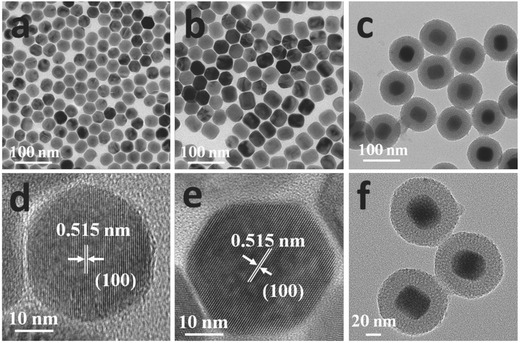
TEM and HR‐TEM images of a,d) core UCNPs, b,e) core–shell UCNPs, and c,f) core–shell UCNPs@mSiO_2_‐Ir.

**Figure 3:**
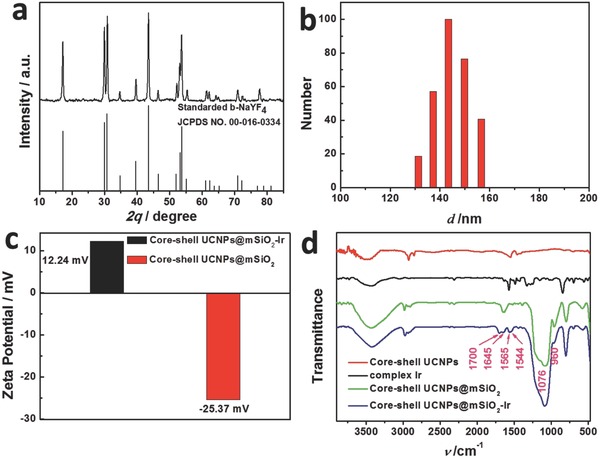
a) X‐ray diffraction pattern of core–shell UCNPs (NaYF_4_: 20 mol% Yb/0.2 mol% Tm@ NaYF_4_) and the standard pattern of β‐NaYF_4_ (JCPDS 00‐016‐0334). b) Dynamic light scattering measurement of core–shell UCNPs@mSiO_2_‐Ir in ethanol. c) Zeta potentials of core–shell UCNPs@mSiO_2_‐Ir and core–shell UCNPs@mSiO_2_ in ethanol. d) Fourier transform infrared spectra of core–shell UCNPs, complex **Ir**, core–shell UCNPs@mSiO_2_, and core–shell UCNPs@mSiO_2_‐Ir. The measurements were conducted at 25 °C.

The prepared hexagonal core UCNPs and core–shell UCNPs were well‐dispersed and uniform with an average diameter of 34 and 38 nm, respectively (**Figure** **2**a,b). Besides, the X‐ray diffraction peaks of UCNPs were in accordance with the standard hexagonal structure of NaYF_4_ (JCPDS NO. 00‐016‐0334) (**Figure** **3**a), which was also confirmed by the high‐resolution transmission electron microscopy (HR‐TEM) images (Figure 2d,e). Uniform mesoporous silica (mSiO_2_‐Ir) layer can be observed around the surface of UCNPs from TEM and HR‐TEM images (Figure 2c,f) of core–shell UCNPs@mSiO_2_‐Ir. The average particle size is 90 nm and the thickness of its outer mSiO_2_‐Ir layer is about 26 nm. Dynamic light scattering (DLS) measurements (Figure 3b) showed that the nanoparticles were dispersed well in solution. Furthermore, stability of the nanoparticles in water has been investigated by DLS (Figure S2, Supporting Information). The size distribution of the nanoparticles showed little change even storing in water for 5 days, indicating that the nanoparticles are quite stable in aqueous solution.

The zeta potential of core–shell UCNPs@mSiO_2_ changed from −25.37 to 12.24 mV when the complex **Ir** was grafted to form the core–shell UCNPs@mSiO_2_‐Ir (Figure 3c). Covalent amide bonds between core–shell UCNPs and complex **Ir** were investigated by Fourier transform infrared (FTIR) spectra (Figure 3d). The bands at 1076 and 960 cm^−1^ can be assigned to the vibration stretching of Si–O, indicating the formation of SiO_2_ layer on the surface of the UCNPs. In comparison with the spectrum of core–shell UCNPs@mSiO_2_, new bands at 1700 and 1645 cm^−1^ of core–shell UCNPs@mSiO_2_‐Ir were attributable to the –C=O stretching vibration of the amide bond and those at 1565 and 1544 cm^−1^ were attributable to the –N–H bending vibration of the amide bond, indicating that complex **Ir** is attached to the nanoparticles through amide bonds. The loading density of the covalently attached complex **Ir** was calculated to be approximately 6345 ± 10 molecules per nanoparticle, which is about 13 times that of the previously reported work,^33^ as determined by UV/visible absorption spectra (Figure S3, Supporting Information). As far as we know, there is no effort to attach molecules to the surface of UCNPs through covalent bonds as much as we do.

### 2.2 Photophysical Properties of Complex Ir and UCNPs

**Figure 4:**
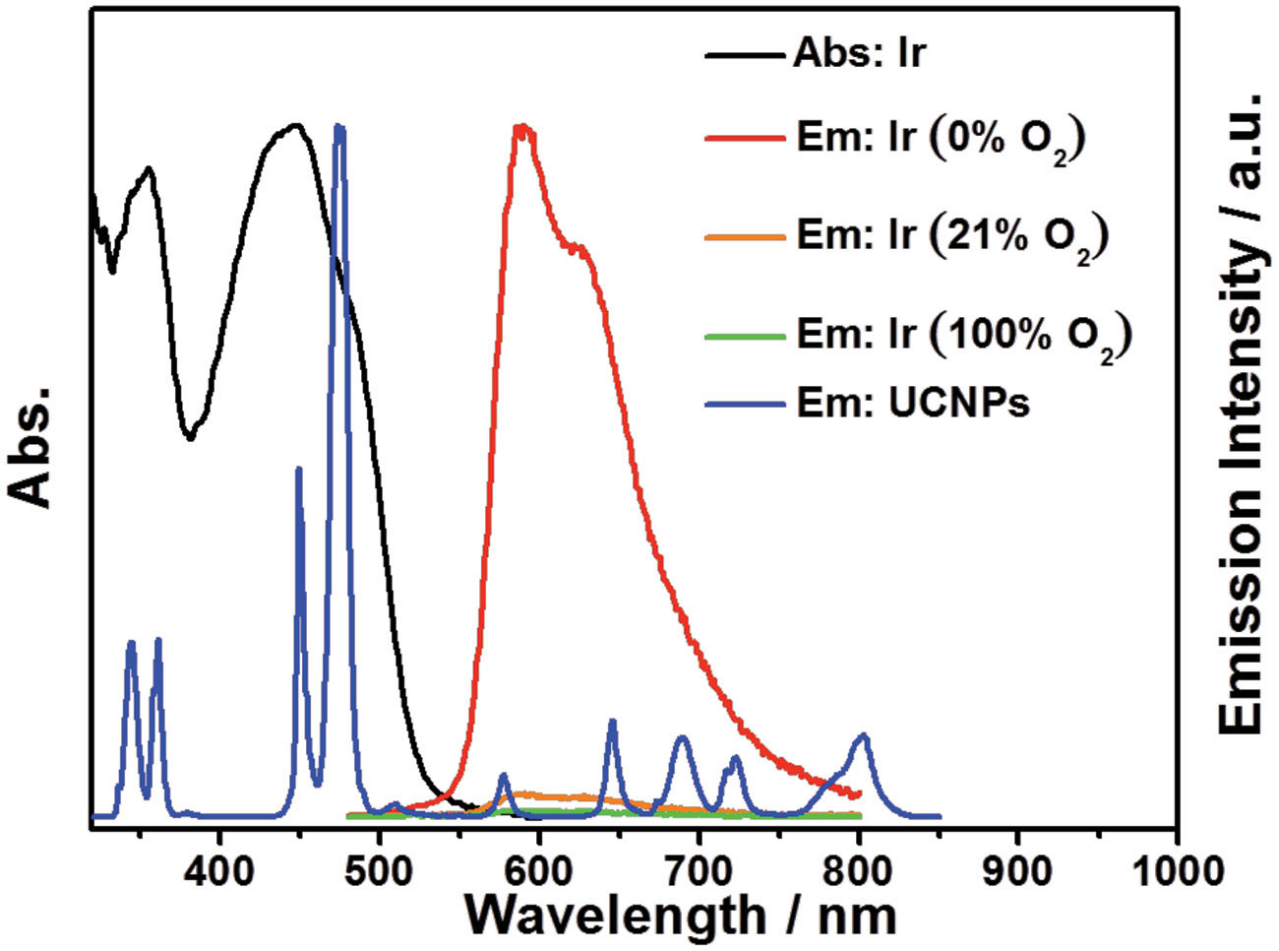
Normalized UV/visible absorption (black solid line) and phosphorescence spectra (red, orange, and green lines) of complex **Ir** under different oxygen concentrations in toluene and upconversion luminescence spectra of NaYF_4_: 20 mol% Yb/0.2 mol% Tm@NaYF_4_ core–shell UCNPs (blue solid line). The spectra were measured at 25 °C.

Complex **Ir** exhibited intense and long‐lived phosphorescence that is highly sensitive toward oxygen quenching (Figure S4a and Table S1, Supporting Information) and the Stern–Volmer plot showed good linear relation (Figure S4b, Supporting Information), which provides the possibility for time‐resolved luminescent imaging of hypoxia. Additionally, there is a good overlap between the absorption bands of complex **Ir** and the ultraviolet and blue UCL bands of NaYF_4_:Yb/Tm UCNPs (**Figure** **4**). Therefore, the ultraviolet and blue UCL of UCNPs with NIR excitation at 980 nm can serve as energy source to excite complex **Ir** through the energy transfer, and result in oxygen‐quenchable phosphorescence.

### 2.3 Oxygen Sensing Performances of Nanoprobe under Upconversion Channel

**Figure 5:**
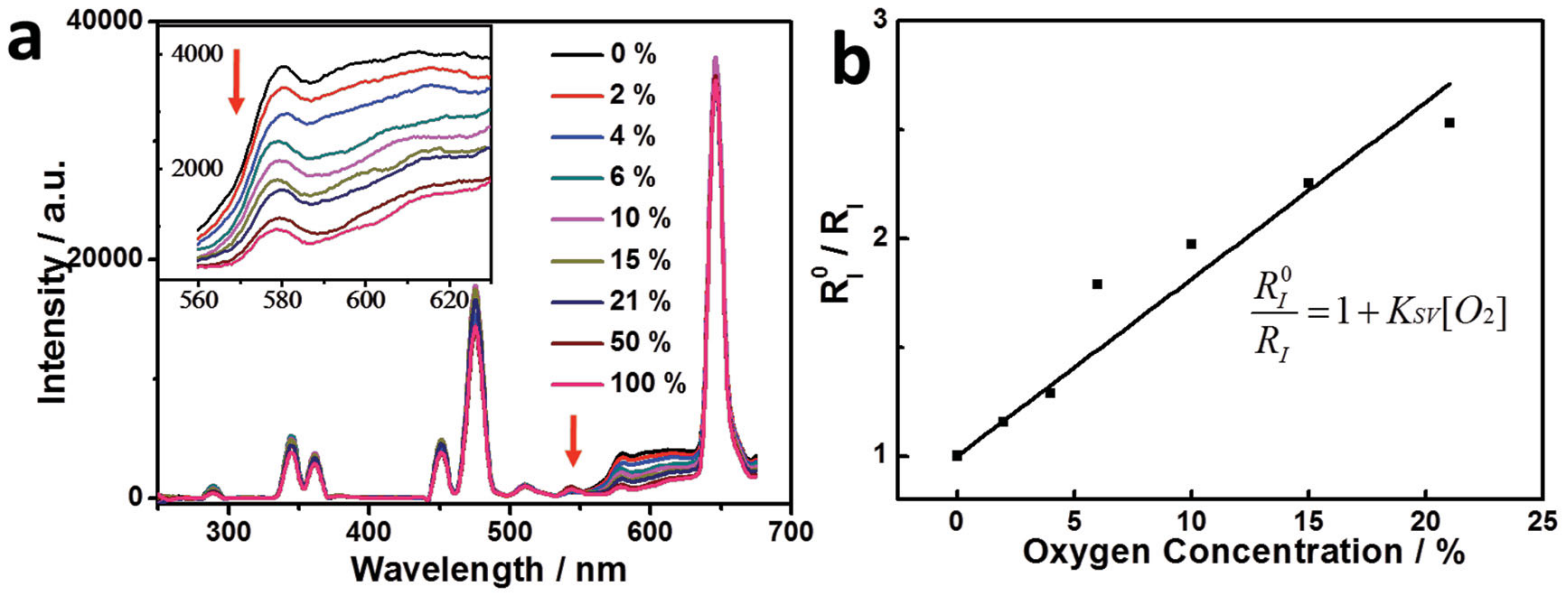
a) Upconversion luminescence spectra of core–shell UCNPs@mSiO_2_‐Ir (1.0 mg UCNPs mL^−1^) under different oxygen concentrations in ethanol and b) the corresponding Stem–Volmer plots (*K*_SV_ = 0.08%^−1^) of the quenching by oxygen. Inset: a) Emission spectra ranging from 560 to 630 nm. *λ*_ex_ = 980 nm, *P* = 2.5 W. The spectra were measured at 25 °C.

To demonstrate the potential of core–shell UCNPs@mSiO_2_‐Ir as a NIR‐excitable oxygen probe, the UCL spectra of the nanoprobe under different oxygen concentrations have been measured under NIR excitation at 980 nm. As shown in **Figure** **5**a, the evident emission from complex **Ir** at 600 nm was observed as a result of energy transfer from UNCPs to complex **Ir**. The emission intensity is very sensitive to oxygen, and it decreased significantly with the increase of oxygen concentration. By contrast, the UCL of UCNPs is insensitive to oxygen concentration (Figure 5a and Figure S5, Supporting Information), which indicates that it can serve as good internal standard in the oxygen sensing application.

Moreover, the quantitative oxygen sensing performances of the nanoprobe under excitation at 980 nm were investigated via a ratiometric method. The ratios of the luminescence intensity of oxygen‐sensitive phosphorescence at 600 nm to oxygen‐insensitive UCL at 477 nm in the absence and presence of oxygen are defined as$R_{I}^{0}$ = $I_{600}^{0}/I_{\mathrm{477}}^{0}$ and *R*_I_ = *I*_600_/*I*_477_, respectively. The following equation can be derived based on the Stern–Volmer equation

(1) $$\frac{R_{I}^{0}}{R_{I}}=1+K_{\mathrm{SV}}[O_{2}]$$

The Stern–Volmer plot of core–shell UCNPs@mSiO_2_‐Ir under excitation at 980 nm revealed a good linear relation between $R_{I}^{0}/R_{I}$ and the oxygen concentration [O_2_], and the *K*_SV_ was calculated to be 0.08%^−1^ (Figure 5b). Thus, the oxygen concentration can be determined based on the *K*_SV_ value from the ratiometric measurements.

To study the energy transfer pattern of our nanoprobe, the UCL spectra of three samples, core–shell UCNPs@mSiO_2_ (1), a mixture of core–shell UCNPs and complex **Ir** (2), and core–shell UCNPs@mSiO_2_‐Ir (3), were measured and compared (Figure S6, Supporting Information). Core–shell UCNPs@mSiO_2_‐Ir displayed reduced UCL than the pure core–shell UCNPs@mSiO_2_, indicating the occurrence of energy transfer, which was defined as total energy transfer. Compared with the mixture of core–shell UCNPs and complex **Ir**, ultraviolet and blue UCL of core–shell UCNPs@mSiO_2_‐Ir decreased and the phosphorescence of complex at 600 nm was enhanced, indicating the possibility of existence of Förster resonance energy transfer (FRET) process besides the emission–absorption–emission process. The total energy transfer efficiency was calculated to be 83%.^41^ The FRET efficiency between ^1^G_4_→^3^H_6_ UCL (477 nm) and complex **Ir** is calculated to be 32% based on the lifetime of ^1^G_4_→^3^H_6_ UCL (477 nm) of core–shell UCNPs@mSiO_2_ (1071 μs) and core–shell UCNPs@mSiO_2_‐Ir (723 μs).^42^

Despite the large loading density of the covalently attached complex **Ir** and high energy transfer efficiency between UCNPs and complex **Ir**, the NIR excited UCL of complex **Ir** around 600 nm was still weak, so that it cannot been detected by confocal microscopy and the corresponding upconversion confocal luminescent imaging was not performed. The low quantum efficiency (*Φ* = 0.05) and weak molar extinction coefficient (*λ*_450_ (*ε*) = 2.99 × 10^4^ m ^−1^ cm^−1^, Figure S7, Supporting Information) may be responsible for this result. Thus, the overlap between emission of donors and absorption of acceptors is not the only consideration in the design of similar system, but also should select acceptors with high quantum efficiency and large molar extinction coefficient.

### 2.4 Oxygen Sensing Performances of Nanoprobe under Downconversion Channel

**Figure 6:**
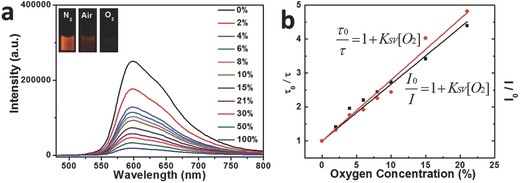
a) Luminescence spectra of core–shell UCNPs@mSiO_2_‐Ir (1.0 mg UCNPs mL^−1^) under different oxygen concentrations in ethanol and b) the corresponding Stem–Volmer plots of the quenching by oxygen (black points stand for the values of *I*_0_/*I* and corresponding *K*_SV_ = 0.17%^−1^, red points stand for the values of *τ*_0_/*τ* and corresponding *K*_SV_ = 0.18%^−1^). *λ*_ex_ = 450 nm. Inset: a) the photos showing luminescence change under different oxygen concentrations when excited at 365 nm. The spectra were measured at 25 °C.

The oxygen‐sensing performances of the nanoprobe under downconversion channel were investigated by emission spectra under excitation at 450 nm. The intensity of phosphorescence located at 600 nm of core–shell UCNPs@mSiO_2_‐Ir decreased with the increase of oxygen concentration (**Figure** **6**a). Simultaneously, the emission lifetime of the nanoprobe also reduced. It is worth noting that the emission lifetimes of the nanoprobe at 0% (4031 ns) and 21% (836 ns) O_2_ concentrations (Table S2, Supporting Information) are much longer than the lifetime of autofluorescence (several nanoseconds), so phosphorescence signal and autofluorescence can be distinguished easily via time‐resolved luminescence imaging technology. Since the emission lifetime cannot be influenced by probe concentration and excitation power, which is different from emission intensity, it is more accurate to determine oxygen concentration by measuring the emission lifetime. The lifetime and luminescence intensity of oxygen‐sensitive phosphorescence at 600 nm in the absence and presence of oxygen are defined as *τ*_600,0_, *τ*_600_, *I*_600,0_, and *I*_600_. The oxygen sensing performance of the nanoprobe can be investigated quantitatively by the following Stern–Volmer equation

(2) $$\frac{\tau_{600,0}}{\tau_{600}}=\frac{I_{600,0}}{I_{600}}=1+K_{\mathrm{SV}}[O_{2}]$$

The Stern–Volmer plots of core–shell UCNPs@mSiO_2_‐Ir under excitation at 450 nm exhibited a good linear relation (Figure 6b), indicating the promising application of core–shell UCNPs@mSiO_2_‐Ir as a hypoxia probe. The *K*_SV_ value obtained from emission lifetime measurement was calculated to be 0.18%^−1^, which was almost equal to that obtained from phosphorescent intensity (0.17%^−1^).

### 2.5 Intracelluar Hypoxia Imaging

**Figure 7:**
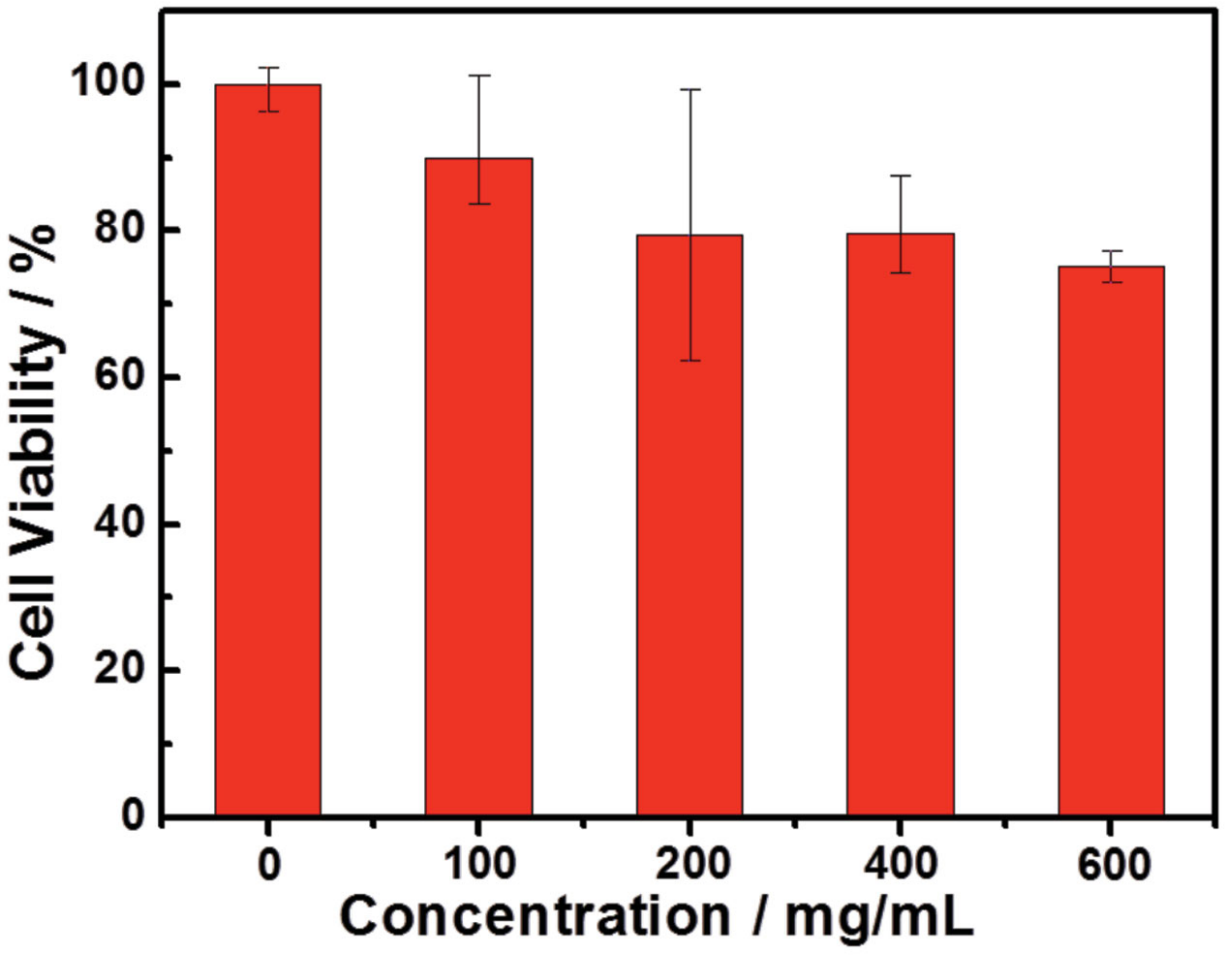
In vitro cell viability of Hela cells incubated with core–shell UCNPs@mSiO_2_‐Ir at different concentrations at 37 °C for 48 h.

**Figure 8:**
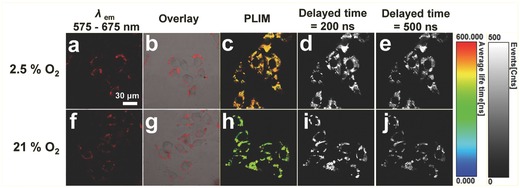
a,b,f,g) Confocal luminescent images, c,h) PLIM images, and d,e,i,j) TGLI images (delayed time = 200 or 500 ns) of living Hela cells incubated with core–shell UCNPs@mSiO_2_‐Ir (200 μg mL^−1^) at 37 °C for 2 h and then incubated at 37 °C and under 2.5% and 21% O_2_ for another 1 h by 405 nm excitation. All the images share the same scale bar of 30 μm. Images were taken at 25 °C.

To investigate the cytotoxicity of the nanoprobe, in vitro cell viability of Hela cells incubated with the nanoprobe under different concentration for 48 h was measured by using methyl thiazolyltetrazolium (MTT) assay (**Figure** **7**). The cell viability remains about 80% when incubated with the nanoprobe at the imaging concentration, indicated the low cytotoxicity of the nanoprobe. Furthermore, the nanoprobe core–shell UCNPs@mSiO_2_‐Ir has been applied to monitoring intracellular oxygen levels in living Hela cells under downconversion channel. Confocal luminescence images showed that the phosphorescence of stained cells at 575 to 675 nm was enhanced when the oxygen concentration reduced from 21% to 2.5% O_2_ (**Figure** **8**a,f). Since the phosphorescence lifetime decreases with the increase of oxygen level, PLIM was used to monitor the change in intracellular oxygen concentration. As shown in Figure 8c,h, the measured intracellular emission lifetime under 2.5% O_2_ concentration (1350 ns) was much longer than that under 21% O_2_ concentration (620 ns), showing evidently different colors (orange for 2.5% O_2_ and green for 21% O_2_) in lifetime images. In addition, TGLI images were also employed to characterize the change of O_2_ concentration (Figure 8d,e,i,j). By exerting a delay time of 200 or 500 ns, which is long enough to remove the short‐lived autofluorescence, the collected TGLI signals can still be clearly observed under both 2.5% and 21% O_2_ concentrations. Besides, the TGLI signals under 2.5% O_2_ concentration were stronger than those under 21% O_2_ concentration, realizing the monitoring of change in intracellular O_2_ concentration via TGLI technique. Thus, on the basis of long‐lived phosphorescence signal, the autofluorescence can be removed and the signal to noise ratio can be improved significantly.

To investigate the mechanism of cellular uptake, Hela cells were incubated with the nanoprobe at 37 °C and 4 °C, respectively. As shown in Figure S8 (Supporting Information), the nanoprobe accumulated more in cells when incubated under 37 °C than 4 °C, indicating that the mechanism of the uptake of nanoparticle by cancer cells could be endocytosis, which relies on energy. To investigate the cell specificity of the probe, the nanoprobe was incubated with Hela cells, human breast cancer cells (MCF‐7), and pancreatic carcinoma cells (PANC‐1), respectively (Figure S9, Supporting Information). The nano­probe can be taken by Hela cells, MCF‐7 cells, and PANC‐1 cells. Considering the nonspecific endocytosis uptake mechanism, there may be litter cell specificity of the nanoprobe. Furthermore, the sub‐cellular distribution of the nanoprobe has been investigated by costained cell imaging. The results showed that the nanoprobe was hardly overlapped with MitoTracker Green or 4,6‐diamino‐2‐phenyl indole (DAPI) (Figure S10, Supporting Information). It can be concluded that the nanoprobe may distribute in the cytoplasm and cell membrane.

**Figure 9:**
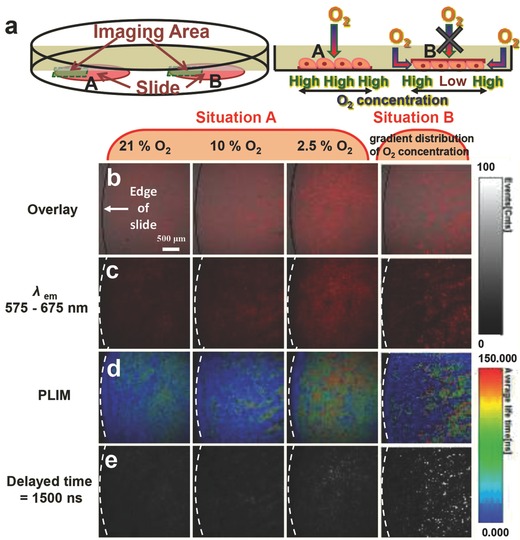
a) Schematic diagram of uniform and gradient O_2_ distribution generated by placing the slide A) under and B) on top of the cells at 37 °C for 3 h after the cells were incubated with core–shell UCNPs@mSiO_2_‐Ir (200 μg mL^−1^) at 37 °C for 2 h, b,c) confocal, d) PLIM, and e) TGLI images of living Hela cells incubated with core–shell UCNPs@mSiO_2_‐Ir (200 μg mL^−1^) upon excitation at 405 nm underuniform O_2_ distribution (21% O_2_, 10% O_2_, and 2.5% O_2_) and gradient O_2_ distribution. All the images share the same scale bar of 500 μm. Images were taken at 25 °C.

Finally, we attempted to visualize the gradient distribution of oxygen concentration in cultured cells under a slide using our oxygen nanoprobe. The gradient distribution of oxygen concentration can be generated by covering a cover glass on the top of cultured cells.^43^, ^44^ The metabolism of cells will consume O_2_, while the slide prohibits oxygen supply from upside, leading to the gradient decrease of O_2_ concentration from the edge to the center of the slide. Herein, the gradient and uniform O_2_ distribution were generated by placing the slide on the top of and under the cells, respectively (**Figure** **9**a). For comparison, ­uniform 21%, 10%, and 2.5% O_2_ distribution have been measured as three references. With the decrease of O_2_ concentration, the phosphorescence at 575 to 675 nm was enhanced significantly (Figure 9b,c). Correspondingly, the emission lifetimes also increased (Figure 9d), and the TGLI signals increased after exerting a delay time of as long as 1500 ns (Figure 9e). These results were consistent with those obtained from the cell imaging experiments. As for gradient O_2_ distribution, the oxygen concentration would decrease from the edge to the center of the slide. Therefore, it can be expected that the intensity of phosphorescence would enhance and the emission lifetime would increase correspondingly, and the TGLI signals would also increase after exerting a delay time as long as 1500 ns. As shown in Figure 9b–e, by comparing the images with the three references, the emission intensity, lifetime, and TGLI signals near the center of slide were similar to those obtained for the reference with uniform 2.5% oxygen concentration. Thus it can be deduced that the oxygen concentration at the center of slide under situation B (Figure 9a) was about 2.5%. Also, it could be deduced that the oxygen concentration in the middle of slide was 10% and that on the edge of slide was 21%. Hence the monitoring of the gradient distribution of oxygen concentration in cultured cells was realized via confocal microscopy luminescence intensity imaging, PLIM imaging, and time‐gated imaging.

## 3 Conclusion

In summary, we have developed an iridium(III) complex‐modified nanoprobe for monitoring oxygen concentration through up and downconversion luminescence. The time‐resolved luminescent imaging technology in downconversion channel and near‐infrared light excitation in upconversion channel were adopted to remove the possible interferences from background autofluorescence. Due to low quantum efficiency and weak molar extinction coefficient of complex **Ir**, upconversion channel of intracellular hypoxia was not realized, while downconversion channel of intracellular hypoxia performed well. Moreover, the nanoprobe has been applied in gradient oxygen distribution successfully which is meaningful to oxygen sensing in tissues with nonuniform oxygen distribution. Considering the highly sensitive oxygen sensing and imaging performances, low cytotoxicity, good water dispersibility, and stability, we believe that our oxygen nanoprobe will be very promising for application in biomedical fields. Further work is directed toward exploiting highly efficient NIR‐excitable oxygen probes for small‐animal imaging.

## 4 Experimental Section

*Materials*: All reagents were purchased from commercial sources and used without further purification. Rate earth oxides Y_2_O_3_ (99.999%), Yb_2_O_3_ (99.999%), Tm_2_O_3_ (99.999%) were purchased from Shanghai Yuelong New Materials Co. Ltd. OA (>90%) and octadecene (ODE) were purchased from Sigma Ltd. All other chemical reagents of analytical grade were used directly without further purification. Deionized water was used to prepare all aqueous solutions.

*General Methods*: NMR spectra were recorded at room temperature by using a Bruker Ultra Shield Plus 400 MHz spectrometer. CDCl_3_ was used as deuterated reagent unless specified. Matrix Assisted Laser Desorption Ionization Time of Flight Mass Spectrometry (MALDI‐TOF MS) was measured on a Voyager DE‐STR. UV/visible absorption spectra were recorded on a Shimadzu UV‐3600 UV–vis‐NIR spectrophotometer. UCL spectra were measured on an Edinburgh FLS‐920 spectrometer with an external 2.5 W adjustable 980 nm semiconductor laser. Photoluminescent spectra were performed with an Edinburgh FLS‐920 spectrometer and emission lifetimes were determined by an Edinburgh FLS‐920 spectrometer with a hydrogen‐filled excitation source. Quantum efficiencies were measured on an Edinburgh FLS‐920 spectrometer with integrating sphere. Oxygen concentration was controlled by a flow counter (HORIBA STEC, SEC‐E40JS, 60 SCCM) during the spectra, lifetime, and quantum efficiency measurements. FTIR spectra were measured using an IR Prestige‐21 spectrometer (Shimadzu) from samples in KBr pellets. X‐ray powder diffraction (XRD) measurements were carried out on a Bruker Smart APEXCCD diffractometer at 40 kV and 20 mA using Cu‐Kα radiation (*λ* = 1.54 Å). Transmission electron microscope (TEM) images were collected on a JEOLJEM‐2100 transmission electron microscope at an acceleration voltage of 200 kV. The as‐prepared samples were dispersed in cyclohexane and dropped onto a copper grid for TEM test. DLS and zeta potential measurements were performed on Brookhaven 90Plus.

*Synthesis and Characterization of Complex Ir—Synthesis of 1‐(4 ‐triphenylamine)ethanone (**1**)*: The synthetic route of complex **Ir** is shown in Scheme S1 (Supporting Information). First, the mixture of ethanol (16.31 mmol) and CH_2_Cl_2_ (25 mL) was stirred at 0 °C for 20 min. Then, acetic anhydride (0.8 mL) was added dropwise and the solution was stirred for another 30 min. After that, the mixture was added dropwise in CH_2_Cl_2_ (40 mL) containing triphenylamine (8.15 mmol) and stirred at 0 °C for 20 min. The light green product was purified on a silica column using hexane/ethyl acetate (30:1, v/v) as the eluent. Yield: 750 mg (32%). ^1^H NMR (400 MHz, [D_6_]CDCl_3_, 25 °C, TMS) *δ*: 7.80 (d, *J* = 8.8 Hz, 2H), 7.32 (t, *J* = 11.2 Hz, 4H), 7.21–7.10 (m, 6H), 6.97 (d, *J* = 8.8 Hz, 2H), 2.51 (s, 3H).

*Synthesis of 2‐(4‐triphenylamine)quinoline (**2**)*: The mixture of 2‐aminobenzaldehyde (2.58 mmol), 1‐(4‐triphenylamine)ethanone (2.58 mmol), sodium hydroxide, and ethanol (30 mL) was stirred at 75 °C for 8 h. After that, the mixture was evaporated to remove the solvent and the residue was purified on a silica column using hexane/dichloromethane (6:1, v/v) as the eluent. Yield: 616 mg (64%). ^1^H NMR (400 MHz, [D_6_]CDCl_3_, 25 °C, TMS) *δ*: 8.19 (d, *J* = 8.4 Hz, 1H), 8.13 (d, *J* = 8.8 Hz, 1H), 8.07–8.00 (m, 2H), 7.85–7.79 (m, 2H), 7.70 (ddd, *J* = 1.2 Hz, 6.8 Hz, 8.4 Hz,1H), 7.50 (ddd, *J* = 1.2 Hz, 6.8 Hz,8.0 Hz, 1H), 7.33–7.24 (m, 4H), 7.22–7.14 (m, 6H), 7.07 (tt, *J* = 1.2 Hz,7.6 Hz, 2H).

*Synthesis of Complex **Ir***: The cyclometalated iridium(III) chloro‐bridged precursor ([Ir(npq)_2_Cl]_2_) with **2** as the cyclometalated ligand was synthesized through the reported method.^45^ The cationic iridium(III) complex was routinely prepared by reacting [Ir(npq)_2_Cl]_2_ precursor with N^N ligand (5‐amine‐1,10‐phenanthroline). First, a mixture of [Ir(npq)_2_Cl]_2_ (0.06 mmol), 5‐amine‐1,10‐phenanthroline (0.15 mmol), and mixed solvent (6 mL, dichloromethane/methyl alcohol, 2:1, v/v) was added in a 50 mL flask and refluxed for 4 h. 5‐amine‐1,10‐phenanthroline was synthesized according to the previous method.^46^ Then, potassium hexafluorophosphate (0.3 mmol) was added into the flask and stirred overnight. The reaction mixture was concentrated under vacuum and purified on a silica column using dichloromethane/acetone (30:1, v/v) as the eluent. ^1^H NMR (400 MHz, [D^6^]DMSO, 25 °C, TMS) *δ*: 8.83 (d, *J* = 8.4 Hz, 1H), 8.48 (d, *J* = 4.8 Hz, 1H), 8.21 (d, *J* = 8.0 Hz, 1H), 8.14–8.02 (m, 5H), 8.02–7.92 (m, 3H), 7.71 (t, *J* = 5.2 Hz 1H), 7.63 (t, *J* = 6.0 Hz 2H), 7.22 (t, *J* = 11.6 Hz, 2H), 7.02 (d, *J* = 9.2Hz, 1H), 6.99–6.76 (m, 24H), 6.72 (s, 2H), 6.52 (t, *J* = 6.8 Hz, 2H), 6.02 (dd, *J* = 11.2Hz, 11.4 Hz, 2H). MS (MALDI‐TOF‐MS): calcd. for C_66_H_47_IrN_7_^+^ 1130.34 [M]^+^; found 1130.83 [M]^+^.

*Synthesis of NaYF_4_*: 20 mol% Yb/0.2 mol% Tm UCNPs (Denoted as Core UCNPs) and NaYF_4_: 20 mol% Yb/0.2 mol% Tm @NaYF_4_ UCNPs (Denoted as Core–Shell UCNPs): Core UCNPs were prepared by a modified solvothermal process according to the reported method.^32^, ^33^ YCl_3_ (0.798 mmol), YbCl_3_ (0.2 mmol), and TmCl_3_ (0.002 mmol) were mixed with oleic acid (6 mL) and octadecene (15 mL) in a 100 mL flask. The mixture was heated to 150 °C to form a homogeneous solution with vigorous stirring under argon atmosphere. After that, the mixture was cooled down to room temperature with the flowing of argon. Then methanol solution (10 mL) containing NH_4_F (148 mg, 4 mmol) and NaOH (100 mg, 2.5 mmol) was slowly added and the solution was stirred at 50 °C for 30 min and then 100 °C for 30 min to remove the methanol. Subsequently, the solution was heated to 290 °C and maintained for 1 h under argon atmosphere before it was cooled down to room temperature. The mixture was precipitated from the solution with ethanol, and collected by centrifugation at 10 000 r min^−1^ for 5 min. The nanoparticles were then washed with ethanol/cyclohexane (9:1, v/v) for three times and redispersed in cyclohexane (5 mL) finally.

For the synthesis of core–shell UCNPs, YCl_3_ (0.5 mmol) was mixed with oleic acid (3 mL) and octadecene (7.5 mL) in a 100 mL flask. The mixture was heated to 150 °C to form a homogeneous solution with vigorous stirring under argon atmosphere. After that, the mixture was cooled down to room temperature with the flowing of argon. Methanol solution (5 mL) containing NH_4_F (74 mg, 2 mmol) and NaOH (50 mg, 1.25 mmol) was added and the solution was stirred at 50 °C for 30 min. Subsequently, the as‐prepared cyclohexane solution of core UCNPs (2.5 mL) was added in the mixture and stirred at 50 °C for another 30 min. Then the mixture was heated to 100 °C for 30 min to remove the methanol and cyclohexane. After that, the solution was heated to 290 °C and maintained for 1 h under argon atmosphere before it was cooled down to room temperature. The mixture was precipitated from the solution with ethanol, and collected by centrifugation at 10 000 r min^−1^ for 5 min. The mixture was then washed with ethanol/cyclohexane (9:1, v/v) for three times and redispersed in cyclohexane (5 mL) finally.

*Synthesis of Core–Shell UCNPs@mSiO_2_*: CTAB (90 mg) and deionized water (20 mL) were stirred mildly at 50 °C for 30 min until the aqueous solution becomes transparent. Then cyclohexane solution of core–shell UCNPs (2 mL, 5 mg mL^−1^) was added in the aqueous solution and stirred at 45 °C overnight to form solution **1**. After that, NaOH (0.2 m, 150 μL), ethanol (3 mL), and deionized water (20 mL) were added in a flask at 50 °C and stirred for 5 min, and then solution **1** (10 mL) was added in the flask dropwise at 60 °C and stirred for another 5 min. Subsequently, TEOS (65 μL) was added dropwise at 70 °C and stirred for 1 h. At last, ethanol (30 mL) was added in the mixture to stop the reaction. The products were collected by centrifugation (11000 r min^−1^, 8 min) and washed for several times with ethanol to remove the residual reactants. Then, the collected nanoparticles were extracted at 45 °C with ethanol solution (30 mL) dissolved with NH_4_NO_3_ (200 mg) overnight to remove the template CTAB. The products were collected by centrifugation (11 000 r min^−1^, 8 min) and washed for several times with ethanol and redispersed in ethanol (5 mL) finally.

*Synthesis of Core–Shell UCNPs@mSiO_2_‐Ir*: First of all, complex **Ir** (12 mg, 0.009 mmol) and 3‐(triethoxysilyl)propylisocyanate (TEPIC) (121 μL, 0.47 mmol) were dissolved in anhydrous tetrahydrofuran (THF) (3.5 mL) in a round‐bottom flask under argon atmosphere. The mixture was heated to reflux at 80 °C in a covered flask for 72 h. After precipitation of the reacted complex **Ir** (TEPIC‐Ir) with cold hexane (15 mL), the precipitate was collected by centrifugation and redissolved in the solution of TEOS (80 μL) and THF (0.8 mL) to form solution **2**. NaOH (0.2 m, 150 μL), ethanol (3 mL), and deionized water (20 mL) were added in a flask at 50 °C and stirred for 5 min, and then solution **1** (10 mL) was added in the flask dropwise at 60 °C and stirred for another 5 min. Subsequently, TEOS (40 μL) was added dropwise at 70 °C and stirred for 40 min, and then solution **2** was added dropwise into the flask and stirred for another 20 min. The products were collected by centrifugation (11 000 r min^−1^, 8 min) and washed for several times with THF to remove the residual reactants. Then, the collected nanoparticles were extracted at 45 °C with ethanol solution (30 mL) dissolved with NH_4_NO_3_ (200 mg) overnight to remove the template CTAB. The nanoparticles were collected by centrifugation (11 000 r min^−1^, 8 min) and washed for several times with ethanol and redispersed in ethanol (5 mL) finally.

Cell Culture: The cell lines Hela were provided by the Institute of Biochemistry and Cell Biology, SIBS, CAS (China). The Hela cells were grown in DMEM (Dulbecco's modified Eagle's medium) supplemented with 10% FBS (fetal bovine serum) at 37 °C with 5% CO_2_. Hela cells were planted on confocal petri dish and allowed to adhere for 24 h before confocal imaging measurement.

*Hypoxia Imaging in Hela Cells*: Hela cells were planted on confocal petri dish and allowed to adhere for 24 h. Before the hypoxia imaging measurements, the old medium was removed and the cells were washed twice with fresh DMEM medium. Then DMEM medium solution contained core–shell UCNPs@mSiO_2_‐Ir (200 μg mL^−1^, 2 mL) was added and the cells were incubated for 2 h at 37 °C with 5% CO_2_. After that, the cells were washed twice with PBS and then fresh PBS (1 mL) was added in the petri dish. For imaging of Hela cells under a certain oxygen concentration, an oxygen concentration‐changeable multigas incubator (Thermo Scientific, SERIES II WATER JACKET CO_2_ Incubator, Model: 3131, S/N: 112620‐1988) was used and the cells were incubated for 1 h under the corresponding oxygen concentration atmosphere before the imaging measurements.

*Confocal Luminescent Imaging*: Confocal luminescence imaging was carried out on an Olympus FV1000 laser scanning confocal microscope equipped with a 40 immersion objective lens. Cells loaded with core–shell UNCPs@mSiO_2_‐Ir were excited by a pulse diode laser head (PicoQuant, PDL 800‐D) with excitation wavelength of 405 nm. The emission was collected from 575 to 675 nm.

*Time‐Resolved Luminescent Imaging*: The time‐resolved luminescent imaging setup is integrated with Olympus FV1000 laser scanning confocal microscope. The luminescent signal was detected by the system of the confocal microscope and correlative data calculation was performed by professional software provided by PicoQuant Company. The light from the pulse diode laser head (PicoQuant, PDL 800‐D) with excitation wavelength of 405 nm and frequency of 0.5 MHz was focused onto the sample with a 40×/NA 0.95 objective lens.

*Cytotoxicity Test*: In vitro cytotoxicity was measured by performing MTT assays on Hela cells. Cells were seeded into a 96‐well cell culture plate at 10^4^/well, under 100% humidity, and were cultured at 37 °C with 5% CO_2_ for 24 h. Different concentrations of core–shell UCNPs@mSiO_2_‐Ir (0, 100, 200, 400, and 600 μg mL^−1^, diluted in DMEM) were then added into the wells. The cells were subsequently incubated for 48 h at 37 °C under 5% CO_2_. Then, MTT (10 μL per well, 5 mg mL^−1^) was added to each well and the plate was incubated for an additional 4 h at 37 °C under 5% CO_2_. The medium was then replaced with dimethyl sulfoxide (DMSO, 150 μL) per well, and OD570 was monitored by an enzyme‐linked immunesorbent assay (ELISA) reader. The following formula was used to calculate the inhibition of cell growth: Cell viability (%) = (mean of *Abs*. value of treatment group/mean *Abs*. value of control) × 100%.

## Supporting information

As a service to our authors and readers, this journal provides supporting information supplied by the authors. Such materials are peer reviewed and may be re‐organized for online delivery, but are not copy‐edited or typeset. Technical support issues arising from supporting information (other than missing files) should be addressed to the authors.

Supplementary

## References

[1] T. Acker , H. Acker , J. Exp. Biol. 2004, 207, 3171. 15299039 10.1242/jeb.01075

[2] T. Yoshihara , Y. Yamaguchi , M. Hosaka , T. Takeuchi , S. Tobita , Angew. Chem. 2012, 124, 4224; 10.1002/anie.201107557 22344795

[3] A. L. Harris , Nat. Rev. Cancer. 2002, 2, 38. 11902584 10.1038/nrc704

[4] C. Murdoch , M. Muthana , C. E. Lewis , J. Immunol. 2005, 175, 6257. 16272275 10.4049/jimmunol.175.10.6257

[5] G. L. Semenza , Annu. Rev. Med. 2003, 54, 17. 12359828 10.1146/annurev.med.54.101601.152418

[6] J. Wang , M. S. Rosello , J. L. Aceña , C. D. Pozo , A. E. Sorochinsky , S. Fustero , V. A. Soloshonok , H. Liu , Chem. Rev. 2014, 114, 2432. 24299176 10.1021/cr4002879

[7] O. S. Finikova , A. V. Cheprakov , S. A. Vinogradov , J. Org. Chem. 2005, 70, 9562. 16268634 10.1021/jo051580r PMC2440654

[8] I. Dunphy , S. A. Vinogradov , D. F. Wilson , Anal. Biochem. 2002, 310, 191. 12423638 10.1016/s0003-2697(02)00384-6

[9] U. Neugebauer , Y. Pellegrin , M. Devocelle , R. J. Forster , W. Signac , N. Moran , T. E. Keyes , Chem. Commun. 2008, 42, 5307. 10.1039/b810403d 18985192

[10] S. J. Zhang , M. Hosaka , T. Yoshihara , K. Negishi , Y. Iida , S. Tobita , T. Takeuchi , Cancer Res. 2010, 70, 4490. 20460508 10.1158/0008-5472.CAN-09-3948

[11] D. B. Papkovsky , R. I. Dmitriev , Chem. Soc. Rev. 2013, 42, 8700. 23775387 10.1039/c3cs60131e

[12] C. F. Wu , B. Bull , K. Christensen , J. McNeill , Angew. Chem. 2009, 121, 2779; 10.1002/anie.200805894 PMC2753190 19253320

[13] A. E. Soini , A. Kuusisto , N. J. Meltola , E. Soini , L. Seveus , Microsc. Res. Tech. 2003, 62, 396. 14601145 10.1002/jemt.10389

[14] S. W. Botchway , M. Charnley , J. W. Haycock , A. W. Parker , D. L. Rochester , J. A. Weinstein , J. A. G. Williams , Proc. Natl. Acad. Sci. USA 2008, 105, 16071. 18852476 10.1073/pnas.0804071105 PMC2570970

[15] A. Martin , A. Byrne , C. S. Burke , R. J. Forster , T. E. Keyes , J. Am. Chem. Soc. 2014, 136, 15300. 25265566 10.1021/ja508043q

[16] A. Grichine , A. Haefele , S. Pascal , A. Duperray , R. Michel , C. Andraud , O. Maury , Chem. Sci. 2014, 5, 3475.

[17] H. B. Sun , S. J. Liu , W. P. Lin , K. Y. Zhang , W. Lv , X. Huang , F. W. Huo , H. R. Yang , G. Jenkins , Q. Zhao , W. Huang , Nat. Commun. 2014, 5, 3601. 24710282 10.1038/ncomms4601

[18] C. Shi , H. B. Sun , X. Tang , W. Lv , H. Yan , Q. Zhao , J. X. Wang , W. Huang , Angew. Chem. Int. Ed. 2013, 52, 13434. 10.1002/anie.201307333 24132981

[19] K. Y. Zhang , J. Zhang , Y. H. Liu , S. J. Liu , P. L. Zhang , Q. Zhao , Y. Tang , W. Huang , Chem. Sci. 2015, 6, 301. 10.1039/c4sc02600d PMC5514630 28757940

[20] Q. Zhao , X. B. Zhou , T. Y. Cao , K. Y. Zhang , L. J. Yang , S. J. Liu , H. Liang , H. R. Yang , F. Y. Li , W. Huang , Chem. Sci. 2015, 6, 1825. 10.1039/c4sc03062a PMC5485888 28694947

[21] H. F. Shi , H. B. Sun , H. R. Yang , S. J. Liu , G. Jenkins , W. Feng , F. Y. Li , Q. Zhao , B. Liu , W. Huang , Adv. Funct. Mater. 2013, 23, 3268.

[22] E. J. New , A. Congreve , D. Parker , Chem. Sci. 2010, 1, 111.

[23] A. V. Kondrashina , R. I. Dmitriev , S. M. Borisov , I. Klimant , I. O'Brien , Y. M. Nolan , A. V. Zhdanov , D. B. Papkovsky , Adv. Funct. Mater. 2012, 22, 4931.

[24] F. Wang , X. G. Liu , Chem. Soc. Rev. 2009, 38, 976. 19421576 10.1039/b809132n

[25] W. Feng , C. M. Han , F. Y. Li , Adv. Mater. 2013, 25, 5287. 23982981 10.1002/adma.201301946

[26] S. Sakadžić , E. Roussakis , M. A. Yaseen , E. T. Mandeville , V. J. Srinivasan , K. Arai , S. Ruvinskaya , A. Devor , E. H. Lo , S. A. Vinogradov , D. A. Boas , Nat. Meth. 2010, 7, 755. 10.1038/nmeth.1490 PMC2932799 20693997

[27] J. Lecoq , A. Parpaleix , E. Roussakis , M. Ducros , Y. G. Houssen , S. A. Vinogradov , S. Charpak , Nat. Med. 2011, 17, 893. 21642977 10.1038/nm.2394 PMC3291110

[28] J. N. Liu , Y. Liu , W. B. Bu , J. W. Bu , Y. Sun , J. L. Du , J. L. Shi , J. Am. Chem. Soc. 2014, 136, 9701. 24956326 10.1021/ja5042989

[29] J. Zhou , Q. Liu , W. Feng , Y. Sun , F. Y. Li , Chem. Rev. 2015, 115, 395. 25492128 10.1021/cr400478f

[30] M. X. Yu , F. Y. Li , Z. G. Chen , H. Hu , C. Zhan , H. Yang , C. H. Huang , Anal. Chem. 2009, 81, 930. 19125565 10.1021/ac802072d

[31] Y. Liu , M. Chen , T. Y. Cao , Y. Sun , C. Y. Li , Q. Liu , T. S. Yang , L. M. Yao , W. Feng , F. Y. Li , J. Am. Chem. Soc. 2013, 135, 9869. 23763640 10.1021/ja403798m

[32] J. N. Liu , W. B. Bu , L. M. Pan , J. L. Shi , Angew. Chem. 2013, 125, 4471;

[33] Y. M. Yang , Q. Shao , R. R. Deng , C. Wang , X. Teng , K. Cheng , Z. Cheng , L. Huang , Z. Liu , X. G. Liu , B. G. Xing , Angew. Chem. 2012, 124, 3179; 10.1002/anie.201107919 22241651

[34] Y. F. Wang , G. Y. Liu , L. D. Sun , J. W. Xiao , J. C. Zhou , C. H. Yan , ACS Nano 2013, 7, 7200. 23869772 10.1021/nn402601d

[35] C. Wang , L. Cheng , Y. M. Liu , X. J. Wang , X. X. Ma , Z. Y. Deng , Y. G. Li , Z. Liu , Adv. Funct. Mater. 2013, 23, 3077.

[36] D. M. Yang , P. A. Ma , Z. Y. Hou , Z. Y. Cheng , C. X. Li , J. Lin , Chem. Soc. Rev. 2015, 44, 1416. 24988288 10.1039/c4cs00155a

[37] X. Zhang , P. P. Yang , Y. L. Dai , P. A. Ma , X. J. Li , Z. Y. Cheng , Z. Y. Hou , X. J. Kang , C. X. Li , J. Lin , Adv. Funct. Mater. 2013, 23, 4067.

[38] C. X. Li , J. Lin , J. Mater. Chem. 2010, 20, 6831.

[39] D. E. Achatz , R. J. Meier , L. H. Fischer , O. S. Wolfbeis , Angew. Chem. 2011, 123, 274; 10.1002/anie.201004902 21031387

[40] F. Wang , J. Wang , X. G. Liu , Angew. Chem. 2010, 122, 7618;

[41] Q. Liu , J. J. Peng , L. N. Sun , F. Y. Li , ACS Nano 2011, 5, 8040. 21899309 10.1021/nn202620u

[42] J. C. Boyer , C. J. Carling , S. Y. Chua , D. Wilson , B. Johnsen , D. Baillie , N. R. Branda , Chem. Eur. J. 2012, 18, 3122. 22344816 10.1002/chem.201103767

[43] W. Piao , S. Tsuda , Y. Tanaka , S. Maeda , F. Liu , S. Takahashi , Y. Kushida , T. Komatsu , T. Ueno , T. Terai , T. Nakazawa , M. Uchiyama , K. Morokuma , T. Nagano , K. Hanaoka , Angew. Chem. 2013, 125, 13266; 10.1002/anie.201305784 24127124

[44] E. Takahashi , M. Sato , Am. J. Physiol. Cell Physiol. 2010, 299, C1318. 20844249 10.1152/ajpcell.00254.2010

[45] N. Zhao , Y. H. Wu , L. X. Shi , Q. P. Lin , Z. N. Chen , Dalton Trans. 2010, 39, 8288. 20694240 10.1039/c0dt00456a

[46] G. F. David , G. Orellana , Helv. Chim. Acta. 2001, 84, 2708.

