# Supplementary material for: A Phosphorescent Iridium(III) Complex‐Modified Nanoprobe for Hypoxia Bioimaging Via Time‐Resolved Luminescence Microscopy
Source: Adv Sci (Weinh). 2015 Jun 25;2(10):1500107. doi: 10.1002/advs.201500107 (PMC5115315; doi:10.1002/advs.201500107)
Supplement: Supplementary file 1 — Supplementary [file ADVS-2-0k-s001.pdf]

Copyright WILEY-VCH Verlag GmbH & Co. KGaA, 69469 Weinheim, Germany, 2015.

ADVANCED SCIENCE
Open Access

Supporting Information

for *Adv. Sci.*, DOI: 10.1002/advs.201500107

A Phosphorescent Iridium(III) Complex-Modified Nanoprobe for Hypoxia Bioimaging Via Time-Resolved Luminescence Microscopy

Wen Lv, Tianshe Yang, Qi Yu, Qiang Zhao, Kenneth Yin Zhang, Hua Liang, Shujuan Liu, Fuyou Li,* and Wei Huang**

Supporting Information

A Phosphorescent Iridium(III) Complex-Modified Nanoprobe for Hypoxia Bioimaging via Time-Resolved Luminescence Microscopy

Wen Lv, Tianshe Yang, Qi Yu, Qiang Zhao, Kenneth Yin Zhang, Hua Liang, Shujuan Liu, Fuyou Li,* and Wei Huang**

W. Lv, Dr. T. S. Yang, Q. Yu, Prof. Q. Zhao, Dr. K. Y. Zhang, H. Liang, Prof. S. J. Liu
Key Laboratory for Organic Electronics and Information Displays & Institute of Advanced Materials (IAM)
Jiangsu National Synergetic Innovation Center for Advanced Materials (SICAM)
Nanjing University of Posts & Telecommunications
Nanjing 210023, China.
E-mail: iamqzhao@njupt.edu.cn
Prof. W. Huang
Key Laboratory of Flexible Electronics (KLOFE) & Institute of Advanced Materials (IAM)
Jiangsu National Synergetic Innovation Center for Advanced Materials (SICAM)
Nanjing Tech University (NanjingTech)
Nanjing 211816, China.
E-mail: wei-huang@njtech.edu.cn
Prof. F. Y. Li
Department of Chemistry and the State Key Laboratory of Molecular Engineering of Polymers and Institute of Biomedicine Science
Fudan University
Shanghai 200433, China
E-mial: fyli@fudan.edu.cn

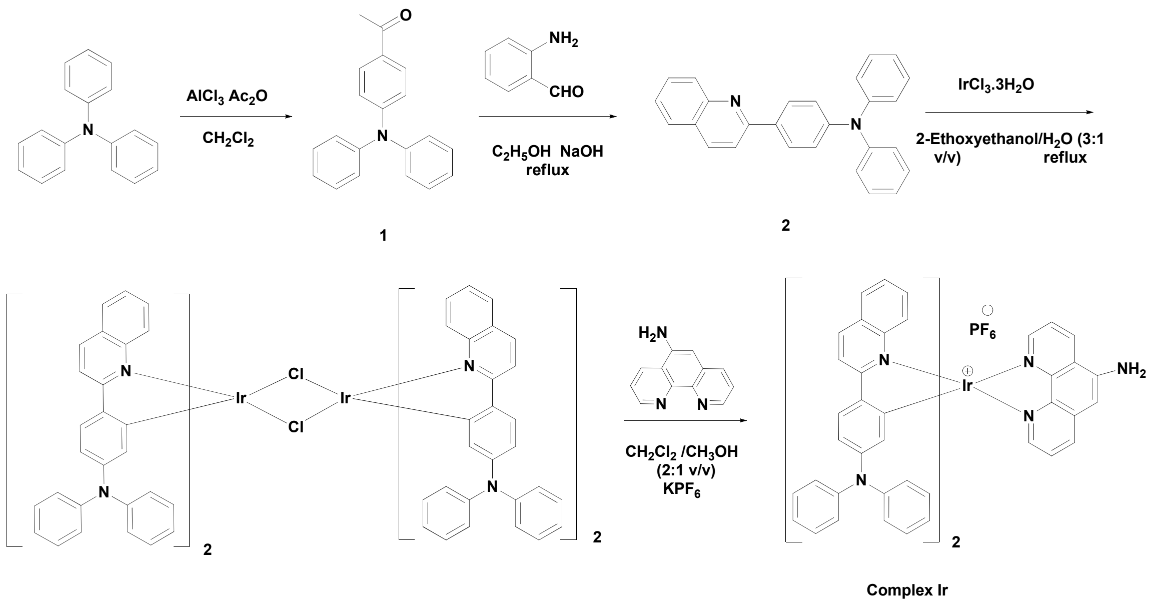

Scheme S1. Synthetic routine of complex **Ir**.

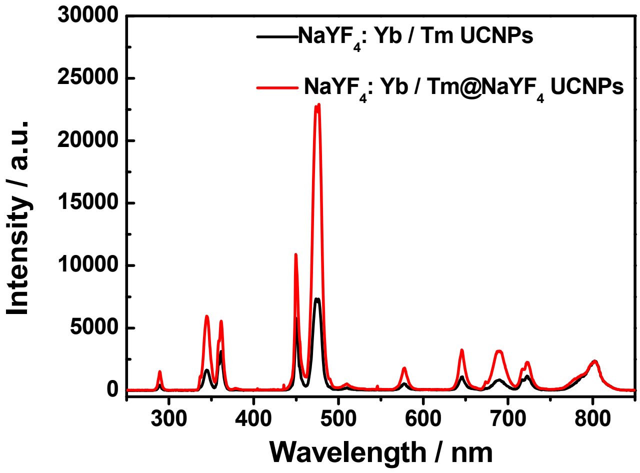

Figure S1. Upconversion luminescence spectra of $NaYF_4$: 20 mol% Yb/0.2 mol% Tm UCNPs (1.0 mg/mL, black line) and $NaYF_4$: 20 mol% Yb/0.2 mol% Tm@$NaYF_4$ core-shell UCNPs (1.0 mg/mL, red line). The spectrum is normalized at 802 nm. λ_{ex} = 980 nm and P = 2.5 W. The spectra were measured at 25 ^oC.

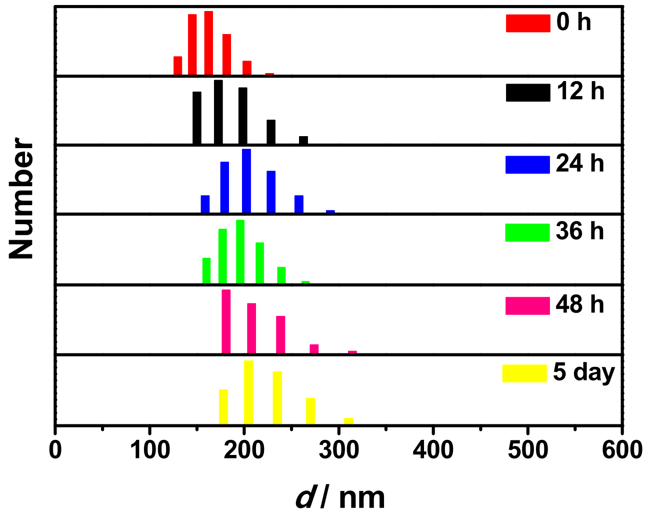

Figure S2. Dynamic light scatter measurement of core-shell UCNPs@mSiO_2-Ir in water. The nanoparticle was stored in water for different time at 25 °C.

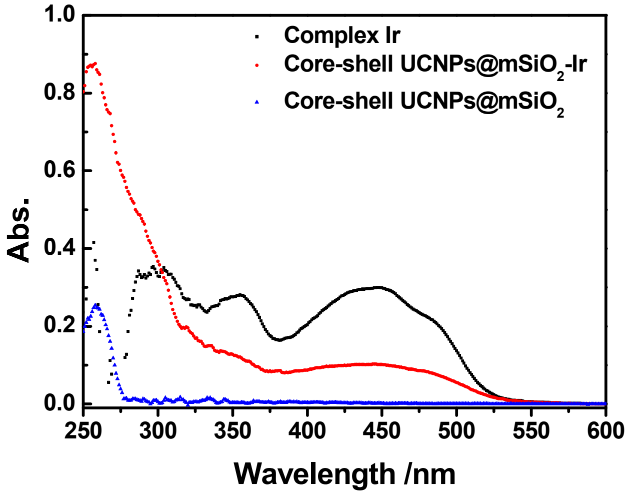

Figure S3. UV/visible absorption spectra of complex **Ir** (1×10^{-5} M) in toluene (black dots), core-shell UCNPs@mSiO_2-Ir (39.1 μg UCNPs/mL) (red dots) and core-shell UCNPs@mSiO_2 (39.1 μg UCNPs/mL) (blue dots) in ethanol. The spectra were measured at 25 °C.

The absorption values at 450 nm were chosen as the original calculation data. The absorption value of core-shell UCNPs@mSiO_2 is too low that we can ignore it. The loading density of complex **Ir** after the reaction was approximately 6345 ± 10 molecules per nanoparticle, which is about 13 times compared to the reported work, as determined by UV/visible absorption spectroscopy. The result was calculated by a method which has been reported previously.[1,2]

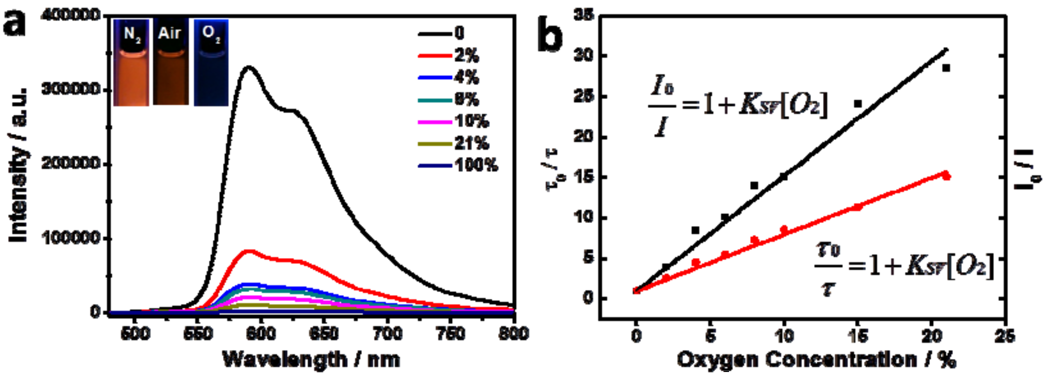

Figure S4. a) Emission spectra of complex **Ir** (1×10^{-5} M) under different oxygen concentrations in toluene (λ_{ex} = 450 nm) and b) the corresponding Stem-Volmer plots of the quenching by oxygen (black points stand for the values of I_0 / I and corresponding K_{SV} = 1.42 $\%^{-1}$, red points stand for the values of τ_0 / τ and corresponding K_{SV} = 0.70 $\%^{-1}$). λ_{ex} = 450 nm. Inset: the photos show change under different oxygen concentration when excited by 365 nm UV light. The spectra were measured at 25 °C.

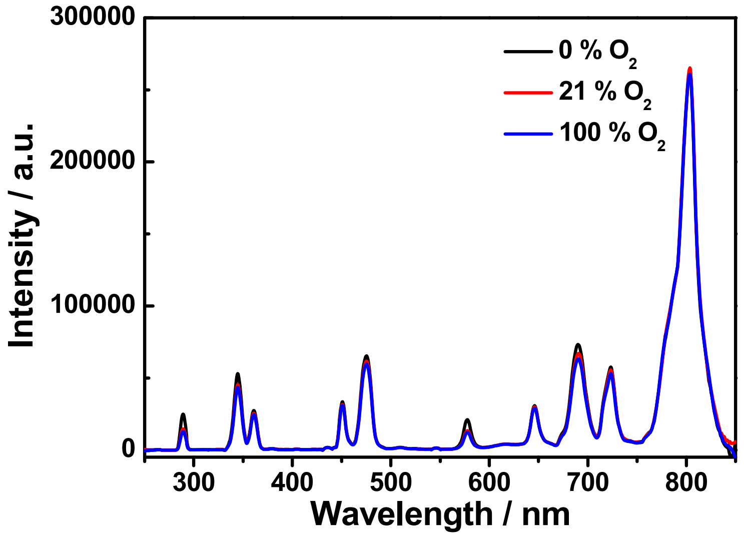

Figure S5. Upconversion luminescence spectra of core-shell UCNPs@mSiO_2 (1.0 mg UCNPs/mL) under different oxygen concentrations in ethanol. λ_{ex} = 980 nm, P = 2.5 W. The spectra were measured at 25 °C.

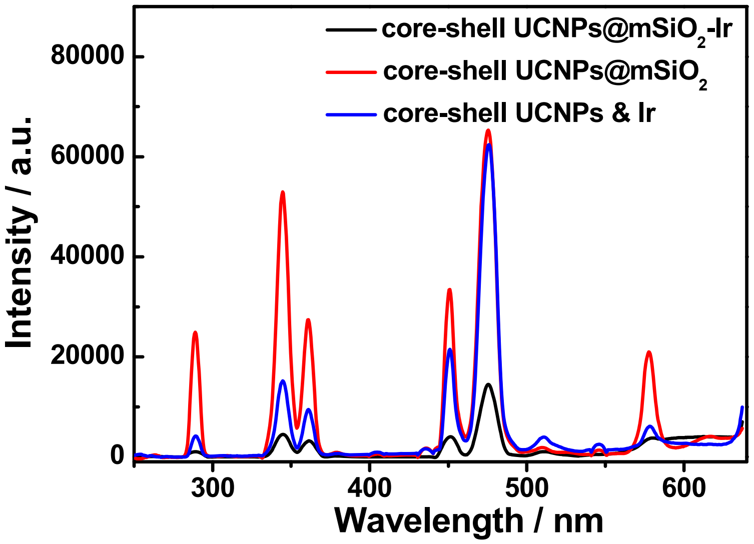

Figure S6. Upconversion luminescence spectra of core-shell UCNPs@mSiO$_2$-Ir (1.0 mg UCNPs/mL, black line) in ethanol, core-shell UCNPs@mSiO$_2$ (1.0 mg UCNPs/mL, red line) in ethanol and the mixture of core-shell UCNPs (1.0 mg/mL) and complex **Ir** (8.73×10^{-5} M, equal to the concentration of complex **Ir** in core-shell UCNPs@mSiO$_2$-Ir, blue line) in toluene under N_2 atmosphere. λ_{ex} = 980 nm, P = 2.5 W. The spectra were measured at 25 ^oC.

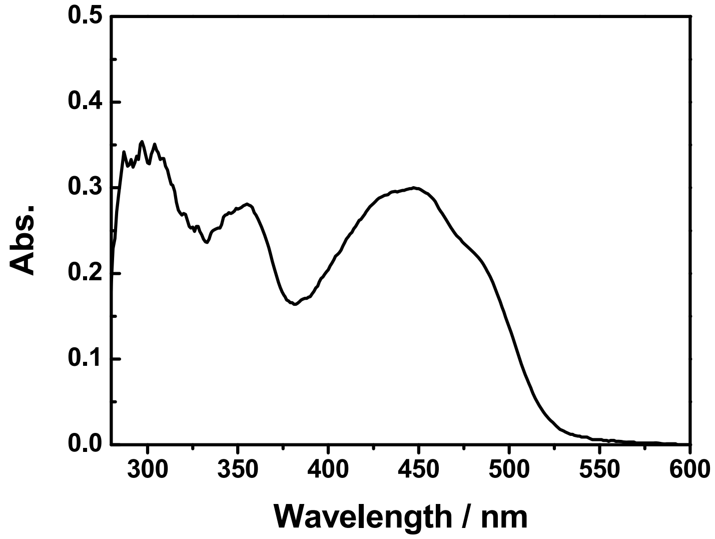

Figure S7. The UV/visible absorption spectrum of complex **Ir** (1×10^{-5} M) in toluene (λ_{450} (ε) = 2.99×10^4 M^{-1} cm^{-1}). The spectra were measured at 25 ^oC.

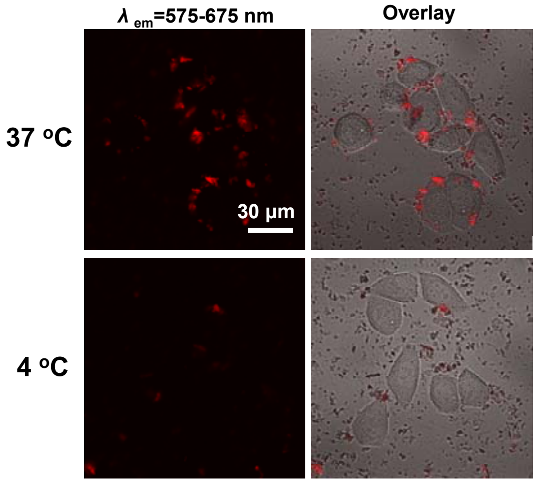

Figure S8. Confocal luminescent images of Hela cells incubated with core-shell UCNPs@mSiO_2-Ir (200 μg UCNPs/mL) at 37 °C and 4 °C for 2 hours by 405 nm excitation. All the images share the same scale bar of 30 μm. Images were taken at 25 °C.

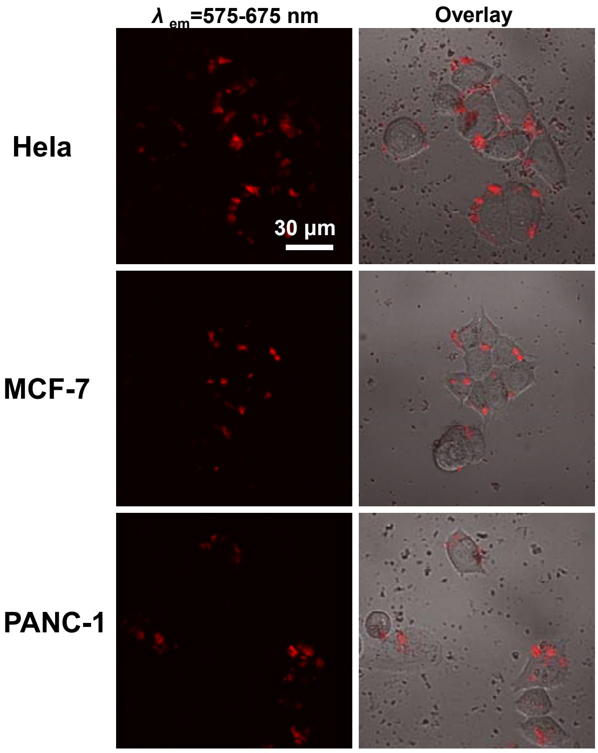

Figure S9. Confocal luminescent images of Hela, MCF-7 and PANC-1 cells incubated with core-shell UCNPs@mSiO_2-Ir (200 μg UCNPs/mL) at 37 °C for 2 hours by 405 nm excitation. All the images share the same scale bar of 30 μm. Images were taken at 25 °C.

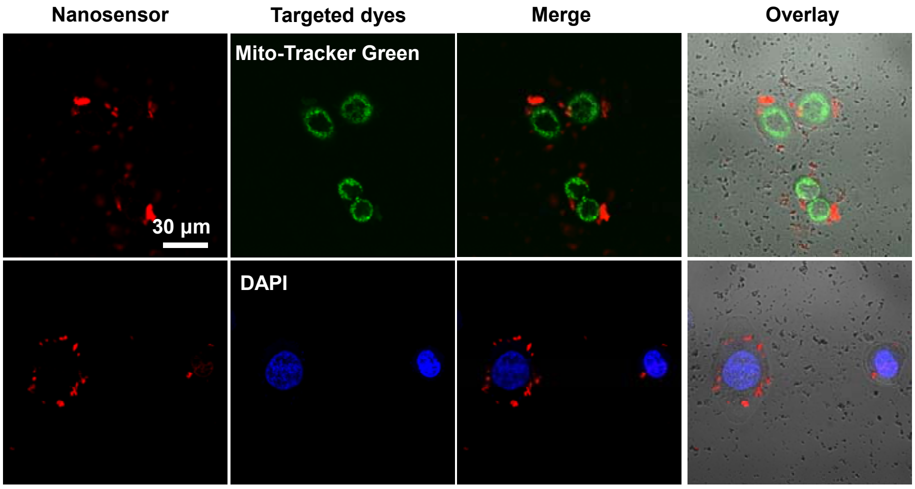

Figure S10. Confocal luminescent images of Hela cells incubated with core-shell UCNPs@mSiO$_2$-Ir (200 μg UCNPs/mL) and co-stained with Mito-tracker Green (200 nM) for and DAPI (10 μg/mL). All the images share the same scale bar of 30 μm. Images were taken at 25 °C. To co-stain with Mito-tracker Green, cells were incubated with Mito-tracker Green (200 nM) at 37 °C for 30 min, and washed with PBS for 3 times. Then the cells were incubated with core-shell UCNPs@mSiO$_2$-Ir (200 μg UCNPs/mL) at 37 °C for 2 h. To co-stain with DAPI, the cells were incubated with core-shell UCNPs@mSiO$_2$-Ir (200 μg UCNPs/mL) at 37 °C for 2 h, and washed with PBS for 3 times. Then the cells were fixed and incubated with DAPI at 25 °C for 10 min.

Table S1. Emission lifetime of complex **Ir** (1×10^{-5} M) under different oxygen concentrations monitored at 590 nm in toluene at 25 °C (λ_{ex} = 450 nm).

Oxygen concentration[a)] [%]	0	2	4	6	8	10	15	21
Average lifetime [ns]	2385	939	530	437	328	278	211	158

[a)]the various oxygen concentration of solution was realized by bubbling corresponding gas mixed with N_2 and O_2 for 10 min.

Table S2. Emission lifetimes of core-shell UCNPs@mSiO$_2$-**Ir** (1.0 mg UCNPs/mL) under different oxygen concentrations monitored at 600 nm in ethanol at 25 °C (λ_{ex} = 450 nm).

Oxygen concentration[a)] [%]	0	2	4	6	8	10	15	21	30	50	100
Average lifetime [ns]	4031	3039	2281	2098	1787	1652	1000	836	685	518	339

[a)]the various oxygen concentration of solution was realized by bubbling corresponding gas mixed with N_2 and O_2 for 10 min.

[1] J. N. Liu, W. B. Bu, L. M. Pan, J. L. Shi, *Angew. Chem.* **2013**, *125*, 4471; *Angew. Chem. Int. Ed.* **2013**. *52*, 4375.
[2] Y. M. Yang, Q. Shao, R. R. Deng, C. Wang, X. Teng, K. Cheng, Z. Cheng, L. Huang, Z. Liu, X. G. Liu, B. G. Xing, *Angew. Chem.* **2012**, *124*, 3179; *Angew. Chem. Int. Ed.* **2012**, *51*, 3125.
